# Cancer related fatigue: implementing guidelines for optimal management

**DOI:** 10.1186/s12913-017-2415-9

**Published:** 2017-07-18

**Authors:** Elizabeth J. M. Pearson, Meg E. Morris, Carol E. McKinstry

**Affiliations:** 1 La Trobe Rural Health School, College of Science, Health and Engineering, La Trobe University, Bendigo, 3552 Australia; 2 La Trobe University, School of Allied Health, Kingsbury Drive, Bundoora, Melbourne, VIC 3086 Australia; 3 Office of Allied Health, College of Science, Health and Engineering, La Trobe University, Melbourne, Australia

**Keywords:** Implementation, Guideline, Cancer-related fatigue, Delphi study, Applicability, Knowledge translation, Consumer, Health professionals

## Abstract

**Background:**

Cancer-related fatigue (CRF) is a key concern for people living with cancer and can impair physical functioning and activities of daily living. Evidence-based guidelines for CRF are available, yet inconsistently implemented globally. This study aimed to identify barriers and enablers to applying a cancer fatigue guideline and to derive implementation strategies.

**Methods:**

A mixed-method study explored the feasibility of implementing the CRF guideline developed by the Canadian Association for Psychosocial Oncology (CAPO). Health professionals, managers and consumers from different practice settings participated in a modified Delphi study with two survey rounds. A reference group informed the design of the study including the surveys. The first round focused on guideline characteristics, compatibility with current practice and experience, and behaviour change. The second survey built upon and triangulated the first round.

**Results:**

Forty-five health practitioners and managers, and 68 cancer survivors completed the surveys. More than 75% of participants endorsed the CAPO cancer related fatigue guidelines. Some respondents perceived a lack of resources for accessible and expert fatigue management services. Further barriers to guideline implementation included complexity, limited practical details for some elements, and lack of clinical tools such as assessment tools or patient education materials. Recommendations to enhance guideline applicability centred around four main themes: (1) balancing the level of detail in the CAPO guideline with ease of use, (2) defining roles of different professional disciplines in CRF management, (3) how best to integrate CRF management into policy and practice, (4) how best to ensure a consumer-focused approach to CRF management.

**Conclusions:**

Translating current knowledge on optimal management of CRF into clinical practice can be enhanced by the adoption of valid guidelines. This study indicates that it is feasible to adopt the CAPO guidelines. Clinical application may be further enhanced with guideline adaptation, professional education and integration with existing practices.

**Electronic supplementary material:**

The online version of this article (doi:10.1186/s12913-017-2415-9) contains supplementary material, which is available to authorized users.

## Background

Evidence-based guidelines have been developed for the management of cancer related fatigue (CRF), yet the feasibility and acceptability of guidelines have not been systematically evaluated [1]. Using the principles of translational science, there is a need to develop strategies to enhance implementation of CRF guidelines. Fatigue is a key concern for a growing number of people living with cancer [2]. It is prevalent during treatment and in advanced cancer [3, 4]. More than 6 months after cancer treatment, approximately 30% of people report moderate to severe ongoing fatigue [5–7]. Fatigue can impair physical functioning and the performance of activities of daily living [8, 9]. It is one of the most frequent unmet needs of people living with cancer [10, 11].

Adoption of clinical guidelines for the management of cancer symptoms such as fatigue can reduce variations in care [12]. Leading cancer organisations such as the National Comprehensive Cancer Network have developed clinical guidelines for CRF [1]. Inconsistency in their clinical application remains [13–18]. In the absence of nationally developed or endorsed CRF guidelines, a critical appraisal concluded the 2015 Canadian Association of Psychosocial Oncology (CAPO) guideline for CRF in adults [8] was appropriate for international use [19]. The CAPO fatigue guideline is targeted towards interdisciplinary oncology teams including psycho-oncology and allied health practitioners, family doctors and palliative care teams [8]. Key guideline recommendations are summarised in Table 1.

**Table 1:** CAPO recommendations for the management of CRF in adults [8]

1. Screen for the presence of cancer related fatigue at specified times or as clinically indicated using a valid quantitative measure
2. If screened positive for fatigue (Score > 2 on a 0–10 numeric rating scale), complete a focused assessment of fatigue and possible medical causes
3. Treat contributing factors and/or refer for further specialist evaluation
4. Evidence is insufficient to recommend pharmacological agents for fatigue at any stage of disease
5. Counsel all patients as is safe to engage in moderate-intensity physical activity for at least 30 min on five or more days of the week
6. All types of physical activity at lower intensity (e.g. walking, yoga) may contribute to decreasing fatigue during and after active cancer treatment
7. All patients are likely to benefit from routine patient education about fatigue self-management
8. Cancer services should promote access to multi-component, group psycho-education programs targeted to self management
9. Referral to experts or fatigue clinics that are trained in cognitive behavioural therapy targeted to fatigue should be offered to patients and survivors with chronic cancer fatigue
10. There is insufficient evidence to advise seeking herbal medicines or acupuncture for treatment of fatigue. Herbal products should be used with caution and patients should discuss their use with the oncology team
11. There is preliminary evidence that mindfulness-based interventions are likely to improve fatigue

The existence of clinical recommendations does not automatically result in adoption and use [20]. Often additional strategies are needed to translate knowledge into practice [20, 21]. Implementing new knowledge involves clinician behaviour change [22] within the context of the organisation and system [23]. Consideration of social, cognitive and motivational factors involving patients, health professionals and organisations is important for implementation [24, 25]. Due to the complexity of guideline implementation, several dissemination and implementation strategies are recommended to enhance compliance [20].

Previous studies identified current clinical practice and selected a guideline for managing cancer related fatigue [16, 19]. The aim of this study was to explore the feasibility and acceptability of the CAPO CRF guideline, to facilitate implementation. The research question was ‘How can the CAPO fatigue guidelines be implemented into clinical practice to reduce cancer related fatigue?’ In particular, we sought to understand whether enhancements to the CAPO fatigue guidelines were needed to facilitate knowledge transfer and implementation. We also explored the systems, training and resources required to enable implementation and rapidly instigate change.

## Methods

A multi-stage mixed-methods design [26] was used to gain an in-depth understanding of perceived barriers and facilitators to implementing the CAPO CRF guideline. Qualitative enquiry using open-ended questions aimed to understand participant views regarding the feasibility of guidelines for managing CRF [27]. The quantitative component was used to determine the degree of agreement with statements related to guideline implementation. This approach was used to increase the meaningfulness and validity of results [28]. After the first stage, content analysis of qualitative data informed the development of the second questionnaire [29]. Qualitative and quantitative data were merged during the final interpretation phase [30].

Many theoretical frameworks and models for evidence translation exist [31–33]. Rogers’ [34] Theory of Diffusion of Innovations underpinned the research and was used in development of survey items. This theory has been previously used to inform adoption of new clinical practices [35], and considers five characteristics of an ‘innovation’ that influence uptake [34]. The Consolidated Framework for Implementation Research (CFIR) constructs within the domains of *intervention characteristics, inner setting and outer setting* guided survey development [36]. These domains were relevant to this broad pre-implementation enquiry. Although not psychometrically validated, the CFIR has been used in several implementation studies [37].

### Setting

The study was based in Australia. Although all Australian residents are eligible to receive public health care [38], issues of distance and workforce can sometimes pose challenges to delivering services. A previous study reported inconsistent use of CRF guidelines across Australia [16]. This suggested a nation-wide approach to this feasibility study was relevant.

### Design

A modified Delphi method was used, enabling participation by stakeholders from various locations and practice settings. Involvement of appropriate stakeholders including consumers is considered an important factor for successful guideline implementation [12, 39]. A key advantage of a Delphi study is that participants from diverse locations may take part anonymously, without domination by individuals in positions of power [40]. The Delphi method is used to achieve a ‘consensus of experts’ where there is a lack of evidence [41]. A Delphi study typically uses a series of two to four questionnaire rounds and aims to generate valid expert opinion on a topic [42]. A modified e-Delphi technique with two primarily electronic survey rounds was utilised to minimise participant drop out rate [43].

### Participants and recruitment

Developers of the Consolidated Framework for Implementation Research (CFIR) recommended that potential barriers and facilitators to implementation should be identified prior to change of practice [36]. Therefore, we sought the views of providers, consumers and administrators of cancer care. End-users of a CRF guideline were identified as general, oncology and rehabilitation health professionals, healthcare administrators and people living with cancer.

Participants were eligible for the study if they had experience with cancer care in one of three groups.

1. Registered medical, nursing or allied health professional with skills and recent experience in cancer care.
2. Professionals in management and policy roles within healthcare organisations.
3. Adults with any cancer diagnosis who have completed primary treatment and have experienced CRF. In addition, exclusion criteria included (a) inability to complete study tasks due to language, cognitive or literacy barriers and (b) professionals not currently or recently practising (within past 6 months).

To enhance the validity of the project a reference panel was appointed to advise on, and oversee the conduct of the study. This panel consisted of the research team and four invited members: an oncology nurse, a medical practitioner, an occupational therapist and a consumer who was recruited via Breast Cancer Network Australia (BCNA) [44].

Reference panel members identified potential health professional (HP) participants using their existing networks, health facility websites, LinkedIn and Google searches, and via a regional oncology team meeting. Snowball sampling was also used as a valid strategy to expand the sample [45]. Invited participants were asked to forward information to appropriate colleagues meeting study selection criteria. Personal email invitations were sent to potential participants, from September to mid-October 2015 and followed up with a reminder email, and/or phone call if required.

For the consumer (CS) recruitment, emails were sent to coordinators of a sample of 22 cancer support and rehabilitation programs, requesting assistance in providing information about the study to their members. The BCNA and Prostate Cancer Foundation of Australia (PCFA) invited members of their survey groups to participate with 827 BCNA women invited in November 2015 and 180 men invited via PCFA to join the second round.

### Participant procedures

Interested participants were sent the Participant Information Statement, consent form and registration form for demographic details. The completed forms were returned to the research team and eligibility and/or consent were verified if needed by telephone. Registered participants were sent reading material, study ID and a survey link or printed survey. Reading material for the first round was a 14-page summary of the CAPO CRF guideline, approved for use in this study by the guideline developer. For the second round, a summary of interim round 1 HP or CS results was provided to the relevant group. Reminder emails were sent. Following each round, a brief update was emailed to all participants. No participants formally withdrew.

Ethics approval was obtained for the project from the La Trobe University College of Science Health and Engineering Human Ethics Committee (HREC: S15–144).

### Development of surveys

Questions in the first survey were drafted based on key literature on implementation research and fatigue guideline implementation, focusing on feasibility and factors associated with evidence uptake. Feasibility indicators of guideline recommendations include compatibility with current practices, acceptability to stakeholders [34] and sufficient detail provided to enact the recommendations (i.e. what to do, for whom) [46]. Elements of the CFIR domain ‘Intervention characteristics’ [36] were used to develop questions regarding perceptions of *relative advantage, complexity, trialability* (potential to pilot test) and *design quality and packaging* (i.e. presentation). ‘Outer setting’ CFIR domain constructs examined *patient needs and resources*, and *external policies and incentives*. The construct most relevant to this study within the ‘Inner setting’ domain was *implementation climate*, using the sub-constructs *tension for change* and *compatibility*.

General attitudes regarding the CRF guideline were evaluated using questions relating to perceived need and trust in the CAPO guideline, satisfaction with current practice and willingness to change [24]. Frequency of implementation or experience of screening, assessment elements and interventions in participants’ health care facilities were used to determine consistency with current practices. Refer to Additional file 1: for surveys.

The reference panel reviewed all draft surveys and reading material for language clarity and face validity. Separate surveys were created for HPs and CS for ease of completion and administration. Surveys were uploaded into Qualtrics® survey software and tested by the research team before distribution. A printed option was available upon request for CS.

The second survey aimed to confirm round 1 findings, and to obtain a deeper understanding of issues with guideline implementation. The research team performed interim analyses of round 1 surveys to enable development of the second round surveys. This included thematic analysis of text data [47] and descriptive numeric data. Data and interim qualitative themes were used to develop choices or statements requiring participants to rate their agreement.

### Data analysis

Qualitative and quantitative data were analysed separately. Descriptive statistics were performed using Statistical Package for Social Scientists™. The research team defined ‘consensus’ a priori to be at least 75% of participants indicating they agree or strongly agree (or disagree/strongly disagree) with a statement. Percentage agreement was commonly used to define consensus in a systematic review, and the median threshold for consensus was 75% [48]. Thematic analysis [47] was applied to text data for open-ended questions using manual methods by two researchers independently (EP and CMK). Themes were refined until agreement was reached. Qualitative and quantitative data strands were mixed at the mid-point to build the second survey, and in the final interpretation stage to develop guideline implementation recommendations. Data were merged in a narrative format with quantitative results woven into the four main qualitative themes [30].

## Results

### Study participants, response rates and survey completion

Email invitations were sent to 95 health professionals and managers (HP group), with 39 registering (45.9% response rate). Nine additional HPs were recruited via snowballing. Three HPs registered but did not complete any part of a survey, and were not included.

A total of 1007 invitations were sent to consumers (CS group) via BCNA and PFCA (for round 2 only). From BCNA, 38 women (4.6% response rate) and from PCFA, 24 men (13.3% response rate) registered. Six enrolled via support groups or snowball method.

Table 2 presents key demographic data, with numbers of participants in each category who completed each survey round. A total of 113 participants completed at least part of one survey: 45 HPs and 68 CS. The participant dropout rate after Round 1 was 27% for HPs and 18% for CS. Although no specific guidelines for acceptable response rates exist for Delphi studies, response rates for Round 2 exceeded the suggested rate of 70% in each round to achieve rigor [42].

**Table 2:** Participants in Delphi study (frequencies)

	Group		Round		
	HP	CS	1	2	Both
Participants: Total	45	68	76	91	60
Health professionals (77% F)	45		40	32	28
Mean age 43.4 SD 11.1 (26–73)					
Allied health professional	19		17	16	15
Nurse	12		10	5	4
Doctor	8		7	4	3
Manager	6		6	6	6
Consumers (64% F)		68	36	59	32
Mean age 61.1 SD 9.0 (36–79)					
Breast		41	33	33	29
Prostate		24		24	
Other		3	3	3	3
Location – Australian state					
Victoria	38	24	46	45	33
New South Wales	5	18	17	21	16
Queensland		14	6	12	5
Tasmania		5		5	
South Australia	1	2	2	2	1
Western Australia		3	3	3	3
Territories (ACT/NT)	1	2	2	4	2
Educational background					
Completed Year 9–11		12	4	12	4
Completed Year 12		13	5	9	4
Bachelor degree	8	20	19	22	14
Masters/PhD/Medical specialty	26	5^a^	25	33	17
Other postgraduate qualification	10	17^a^	22	25	20
Unspecified		1		1	

^a^ Masters and above not recorded for all consumers

Disciplines among the HPs and managers included two general practitioners and six specialist doctors, 14 occupational therapists, 13 nurses, three physiotherapists, two exercise physiologists, two dieticians and one each of psychologist, social worker and health administrator. Average time since qualification was 19.72 years (SD 11.25) and average years in oncology practice were 13.63 (SD 9.87). The median proportion of oncology caseload was 98%. Seven HPs (16%) were from rural or regional locations.

The CS participants lived in metropolitan (60%), rural or regional (35%) and remote areas (3%). Consumers reported experiencing CRF during treatment (73%), after treatment (75%) and/or currently (66%). The average time since most recent cancer diagnosis was 3.48 years (SD 2.89) and 25% reported they currently received ongoing treatment.

### Feasibility of the CAPO fatigue guidelines

Data presented in Table 3 indicates that both HP and CS participants perceived a need for a CRF guideline, but HPs were more cautious than CS were regarding its net benefits. Proportions meeting the *a priori* defined criterion for consensus of 75% agreement are shown in bolded text.

**Table 3:** Survey 1 - General attitudes toward CAPO CRF guideline

Statement	Survey	N	Agree (n)^a^	Agree (%)
There is a need for clinical guidelines for management of cancer-related fatigue (CRF) tailored for the Australian context	HP1	43	34	**79.1**
	C1	63	52	**82.5**
The benefits of the CAPO guideline outweigh the costs, inconvenience or discomfort	HP1	40	24	60.0
	C1	32	28	**87.5**
I am satisfied with current approaches to CRF management at my workplace/health care facility^b^	HP1	48	25	52.1
	C1	97	46	47.4
I would adopt or trial the CAPO CRF guideline in its current form	HP1	40	31	**77.5**

^a^ Agree or strongly agree; ^b^ Participants answered for up to 3 health facilities; bolded figures indicate *a priori* definition of consensus was met

Results in Table 4 suggested that CAPO CRF guideline elements were regularly implemented approximately one-third of the time. Although perceptions of HPs varied, the majority considered there was sufficient detail to implement most guideline elements.

**Table 4:**
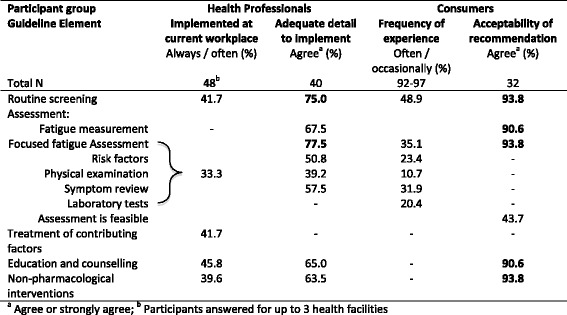
Survey 1 - Indicators of feasibility of guideline elements

Four main themes emerged from the qualitative data analysis. Statements within each theme that met the consensus criterion are presented. Data for items that did not reach consensus are available in Additional file 2.

- A need to balance simplicity with adequate detail in the guideline
- Defined roles for knowledgeable health professionals
- Integrate CRF management with existing practices
- Consumer focused care: ‘for each person it’s a personal thing’ (C81)

#### Theme 1: A need to balance guideline simplicity with detail

- *Layout and presentation.*
  Several participants commented that the fatigue guideline was too long and too detailed for optimal clinical utility. These participants favoured brevity, with tables and flow charts to enable use by busy clinicians: ‘Visual layout could be enhanced for greater user-friendliness’ (senior occupational therapist (OT) A49). A general practitioner (GP) commented: ‘No GP is going to read the whole lot. Make it more accessible – shorter – few pages, summaries’ (M45). Consumer support for a CRF guideline version in lay language was illustrated by one comment: ‘When undergoing cancer treatment there are enough issues to be faced without having further complicated instructions’ (C80).
- *Guideline content.*
  Some HPs considered the CAPO guideline recommendations too complicated, while others reported insufficient detail to enact: ‘I found the guidelines more about assessing the literature rather than about what to do’ (medical oncologist M56). Two OTs disagreed: ‘Increase detail and specificity as far too broad to provide clinical guidance’ (A44) and ‘I think currently there is too much detail for the reality of working on an acute oncology ward’ (OT A48). Data supported these perceptions; particularly related to implementing physical examination and risk factors (Table 4). Some HPs were concerned about what to do after assessment, suggesting inclusion of decision rules in an algorithm may increase utility. A nurse commented ‘You can conduct assessment but it’s difficult to determine what to do after that’ (N33).

Participants proposed enhancements to the CAPO guideline presentation and content, which were evaluated in the round 2 surveys. Those meeting the consensus criterion are presented in Table 5. See Additional file 2: for full results.

**Table 5:** Statements meeting consensus criterion related to themes 1 & 2

Theme 1: A need to balance simplicity with adequate detail in guideline	Survey	N	Agree (n)	Agree (%)
The guideline should be written for consumers in lay language with a simple rationale for each recommendation	C2	59	59	100
A recommended valid patient self-report measure for fatigue and related constructs would be helpful to monitor progress across settings	HP2	32	32	100
A self-report questionnaire (such as the Brief Fatigue Inventory) that measures fatigue severity and impact could be useful as part of focused fatigue assessment and history taking	HP2	32	30	93.7
Referral pathways with capacity for local adaptation would be clinically useful	HP2	31	29	93.5
A screening hand-out could contain simple information including diagrams about how to rate your fatigue level, and what to do for different levels of fatigue	C2	59	55	93.2
Information about how to get help for fatigue management in my local area would be useful	C2	59	54	91.5
The guideline should contain links to additional detail about specific contributing factors such as medications, electrolyte levels, nutritional parameters and rate of physical activity change	HP2	32	28	90.3
Information and education about fatigue should be offered at different levels of detail (e.g. basic, standard, detailed)	C2	59	53	89.8
A short-list of accessible valid fatigue screening tools would be helpful	HP2	32	28	87.5
The guideline should contain links to additional detail about physical examinations and significance of findings	HP2	31	27	87.1
Appendices with details of assessments/interventions	HP2	31	25	80.6
I find diagrams such as flow-charts helpful to understand and learn new information	C2	59	46	78.0
Stratified assessment guide	HP2	31	24	77.4
Theme 2: Define roles for knowledgeable health professionals	Survey	N	Agree (n)	Agree (%)
I want to know which health professional is overseeing and monitoring my fatigue levels and supporting me	C2	59	58	98.3
A practice nurse (or other designated professional) could screen for tachycardia, shortness of breath and signs of nutritional deficiencies (oral) and anaemia (eyes) and refer to the appropriate professional for further assessment	HP2	31	30	96.8
Gait, posture, muscle wasting and range of motion would ideally be assessed by a relevant health professional; if appropriate	HP2	31	30	96.8
Make it real. I would like health professionals to know about how fatigue has affected real people like me	C2	59	57	96.6
All clinicians should be able to screen for fatigue	HP2	32	30	93.8
On-line education about managing fatigue should be available to all health professionals involved in my cancer care	C2	59	55	93.2
Determination of which HPs should take responsibility for assessments, interventions and follow up would improve consistency of practice	HP2	31	27	87.1
What is your preferred method/s of learning about assessment and management of CRF? Interactive website	HP2	31	24	77.4

#### Theme 2: Who does what - define roles for knowledgeable health professionals

A major theme in the first surveys was a need to define and designate HP roles in CRF management: ‘All elements are so dependent on the team, the team members and their availability and expertise. It is difficult to know what is being provided, by whom’ (OT A26). An ambulatory care manager suggested: ‘Clarify which practitioner would undertake these assessments and pathway for escalation’ (G17). Consumers wanted to know: ‘who would be doing the assessment … in a multidiscipline team who would be allocated to monitor to take the confusion out of the picture?’ (C83) and ‘who is ultimately responsible for referring the patient, follow up etc’ (C92). Consumers and HPs wanted information about local expertise in CRF management: ‘Would be nice to have the guidelines altered to have who at our local institution can do each aspect of this and fax/phone numbers’ (palliative care physician M57); ‘Information about classes for exercise, yoga, or meditation that are linked up to the hospital and suitable for recovering patients’ (C72). Table 5 presents consensus data related to defined roles and health professional training.

A sub-theme from both participant groups was access to HPs with expertise in CRF management: ‘Regional areas are severely lacking in professionals who understand how hard it is to cope with fatigue’ (C81); ‘access to experienced allied health is a key challenge’ (oncologist M05). Several CS commented that they had not received helpful advice from their HPs regarding fatigue management, suggesting a lack of up-to-date knowledge about CRF: ‘I was told oh that is normal go with the flow, in other words put up and shut up’ (C86), ‘sometimes it feels like its guess work’ (C122) and ‘it was only after many months of feeling totally drained that I approached the doctor to see what was wrong with me’ (C80). A nurse coordinator stated: ‘health professionals may require specific education themselves so that they truly understand CRF … and then be able to provide appropriate education and interventions’ (N03). Consumers echoed the need for HP education: ‘train the medicos!’ (C70); ‘I think once a GP has a patient diagnosed with a particular cancer they should receive online training (that they only have to do once) so they learn what could happen during that person’s journey’ (C122).

#### Theme 3: Integrate CRF management with existing practices

The third main theme recognised a need to integrate CRF management with current practices and systems: ‘If we are going to screen patients regularly it needs to be quick and built into existing processes’ (oncology nurse N38). A cancer nurse consultant stated: ‘assessment and care maps need to be integrated within existing assessment and screening tools that are used within organisations’ (N41). Consumers wanted fatigue management as a routine part of cancer care, with screening, assessment and education during regular appointments or treatments such as chemotherapy: ‘Make sure the assessment process dovetails with a patient’s existing appointments so that extra time and effort is not needed’ (C68); ‘Often chemo involves sitting in a chair for a few hours so it’s a perfect opportunity to present information’ (C19); and ‘I think including a fatigue assessment in a routine appointment would add to the feeling of being properly managed’ (C77).

Health professionals were concerned about time and cost in the context of overall patient care: ‘How long the assessment takes: I would need to be sure that an extensive, fatigue only assessment was going to have good outcomes versus a multi-symptom assessment tool’ (nurse unit manager N33). Opinions in this theme were somewhat divergent, with fewer items reaching consensus. Refer to Table 6 for consensus statements related to integration of CRF management with current practice.

**Table 6:** Statements reaching consensus regarding themes 3 and 4

Theme 3: Integrate CRF management with existing practices	Survey	N	Agree (n)	Agree (%)
Fatigue management should be a part of routine cancer services	C2	59	57	96.6
Once clinicians identify moderate to severe fatigue they should seek advice and/or refer for comprehensive assessment	HP2	32	31	96.9
Ask me about my fatigue level during routine appointments	C2	59	48	81.4
A self-assessment for patient to identify issues would be time-efficient for clinicians	HP2	32	26	81.3
Applying standardised diagnostic criteria for CRF is useful in the clinical setting to distinguish CRF from other types of fatigue	HP2	32	26	81.3
Theme 4: Consumer-focused care	Survey	N	Agree (n)	Agree (%)
Essential time points for fatigue screening				
At diagnosis	HP2	32	24	75.0
At diagnosis or start of treatment as baseline	C2	59	48	81.4
At end of a treatment course	C2	59	52	88.1
During routine assessment before each new treatment	C2	59	52	88.1
After hospitalisation or changed health status	C2	59	51	86.4
3 months post treatment	C2	59	50	84.7
At annual check up	C2	59	48	81.4
It is essential to be made aware of the possibility of fatigue and how to measure it, when you are first diagnosed with cancer	C2	59	53	89.8
Information and education about fatigue should be offered at different levels of detail (e.g. basic, standard, detailed)	C2	59	53	89.8
Access to individual or group education about fatigue supported by written material is important to me	C2	59	53	89.8
It is important to me to have some say in when, where and how I am assessed if I have moderate to severe fatigue	C2	59	52	89.8
I would like to be given the choice of doing a paper, electronic or verbal questionnaire to assess my fatigue	C2	59	50	88.8
I would prefer a longer appointment for fatigue assessment compared to extra visits	C2	59	46	78.0
More detailed information about fatigue prevention can come once treatment has started.	C2	59	45	76.2
One of the five most important factors that would encourage you (CS) to adopt the CAPO guideline: If my health professional promoted its use	C1	32	24	75.0

To enable implementation of the CAPO fatigue guideline, some participants noted that changes to policy and remuneration systems would be needed. ‘Successful implementation of this guideline would be resource-intensive at our health service, therefore significant changes to our current model of care would be required’ (physiotherapist/Improvement Facilitator G16). An ambulatory care manager highlighted consideration of fatigue in the context of other needs: ‘We need to tackle assessment of unmet needs more broadly for all patients at the start and end of treatment’ (G17). A GP had remuneration concerns: ‘Be paid adequately for the time to be able to undertake the comprehensive assessment and provide advice on management’ (M45). Endorsement by a peak body or organisation was listed among the top 5 factors influencing guideline use (Round 1).

#### Theme 4: Consumer focused care - ‘for each person it’s a personal thing’ (C81)

Several CS noted that CRF is an individual experience and that management needs to be tailored to fatigue related limitations. These included limited stamina, difficulty with a range of cognitive tasks, time demands and travel to appointments. One participant stated: ‘As many cancer survivors have to balance their family lives as well as work these (fatigue management) strategies need to be individually tailored, and effective enough for people with cancer to devote their time to’ (C35). Some HPs noted that tailoring for people at different stages of cancer was needed: ‘In palliative care CRF can be overlooked as just part of the process and distress around this symptom is not always identified’ (OT A30); ‘From a practical perspective, the guidelines could be adapted for people currently undergoing treatment … vs. those who have completed treatment’ (OT A18).

Some CS noted that emotional status might impact the CAPO fatigue guideline’s feasibility: ‘I think people undergoing cancer treatment are bombarded with so much information, so many tests, so much treatment all for their benefit by so many practitioners, it is overwhelming, confusing and downright scary. Also, depending on your stage and severity of cancer, your level of support you are very aware of your mortality and are very vulnerable’ (C90). Several CS had experienced a lack of HP understanding: ‘I would like a professional to really listen to me when I tell them that I am fatigued! My GP is great, but busy’ (C70); ‘someone who listens but doesn’t judge’ (C81). Table 6 includes consensus statements related to consumer-focused care.

Early and frequent screening and awareness about fatigue was important for CS, in contrast to HPs who only agreed on one essential time point. Preferences for mode of questionnaire and consumer education differed among CS.

### Recommended implementation strategies

**Fig. 1:**
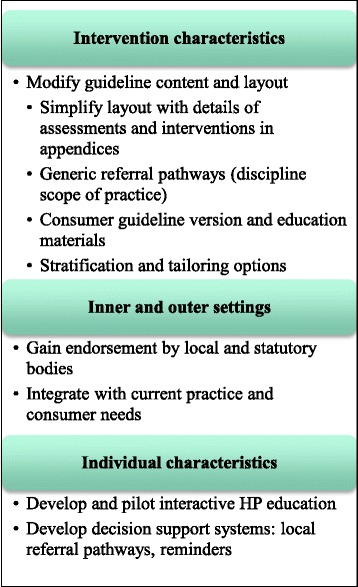
Recommended implementation strategies

The results indicate that several implementation strategies could enhance the feasibility of the CAPO fatigue guideline, listed in Fig. 1 according to CFIR domain [36].

## Discussion

Participants in this study perceived the CAPO CRF guideline to be potentially beneficial but lacking clinical utility in its current form. Most participants were in favour of a simplified format, added detail about procedures and HP role designation. Consumers requested a lay version of the guideline. A systematic review of guideline implementation strategies reported that the complexity of guideline content and format inversely influenced uptake [20]. Several researchers have concluded that, for optimal uptake, guideline recommendations should be readable and relevant to the clinician [49], and include specific target behaviours: what to do, by whom [50, 51]. Kastner and colleagues [39] hypothesised that availability of multiple versions of guidelines for different purposes may increase uptake.

A need to integrate CRF management with existing practice was identified, consistent with recommendations of recent studies [16, 52]. Compatibility with existing practices and workplace culture is an important attribute influencing adoption of an innovation or guideline [34, 36, 53]. Health professional participant concerns regarding adequacy of resources (time, staff, programs) for implementing the CAPO fatigue guideline were consistent with findings of a systematic review of perceived barriers to delivering psychosocial cancer care [54].

Participants perceived the need for accessible HP education regarding management of CRF. This concurred with findings of previous studies [17, 25, 55]. Capacity building including HP training is deemed integral to an implementation plan [36]. However, a systematic review reported that passive education alone is insufficient to ensure translation to practice, and interactive or outreach education is reported to be more effective than passive modalities [20].

Consumer involvement in this study highlighted the need for accessible, consumer-focused CRF management tailored to functional capacity. We found that guideline efficiency and effectiveness were important to CS with limited energy, as well as for HPs. However, evidence of feasibility and outcomes of CRF guideline use is currently limited. A retrospective study at a CRF clinic that utilised the 2007 National Comprehensive Cancer Network (NCCN) guidelines reported lowered fatigue levels [56]. Improvements in fatigue and functioning were reported in a randomised controlled trial of symptom management for patients with advanced cancer and CRF, one element of the NCCN fatigue guideline [57]. Details of costs and outcomes of using the full CRF guideline have not yet been reported.

According to the UK Medical Research Council parameters [58], the CAPO fatigue guideline is a ‘complex intervention’. Full implementation involves multiple disciplines, complex assessment and a range of fatigue management interventions [8]. Complex interventions such as clinical guidelines require pilot testing to determine the feasibility of delivery within a given health care context [58]. Our results suggested that a feasibility study is appropriate. An implementation trial in several phases would provide an opportunity to trial and refine elements, gather evidence of the guideline’s impact and identify resource needs and costs. Additional implementation strategies reported to be effective could be considered, such as identifying opinion leaders, engaging change agents and monitoring satisfaction and impact of guideline use [20].

This study had several strengths and limitations. A broad examination of the CAPO CRF guideline’s applicability was achieved using several theoretical approaches within a mixed methods study [33, 59]. The Delphi survey method enabled a diversity of views to be captured and triangulation of findings [42]. It is uncertain whether the response and attrition rates are acceptable due to lack of guidelines relating to this [42]. Data gathered from in-depth interviews or focus groups may have identified other aspects of guideline feasibility than was possible using written responses.

Involvement of the reference panel to oversee study procedures increased study rigor. Expansion of the survey panel with additional male consumers for Round 2 achieved an acceptable gender balance and increased generalisability of the results. Despite this, the majority of HPs on the survey panel were located in one Australian state, and almost all CS had a diagnosis of either breast or prostate cancer, so results require verification in other locations and cancer types. Recruitment by email, limited access to email addresses of relevant HPs and recruitment of CS via third parties reduced the scope of invitees. Although the response rate of HPs was satisfactory, busy invitees may have overlooked emails.

## Conclusion

Translation of research regarding optimal management of cancer related fatigue into clinical practice can be enhanced by adoption of valid clinical guidelines. This study found that health professionals, people with cancer and administrators believed the CAPO fatigue guidelines to be useful. Modifying the guideline presentation, health professional education, and integration with existing practices could further enhance implementation.

## Additional files

Additional file 1: Survey questionnaires HP1, C1, C2 and HP2. (DOCX 311 kb)

Additional file 2: Results of all quantitative survey questions. (DOCX 119 kb)

## Abbreviations

BCNA: Breast Cancer Network Australia
CAPO: Canadian Association for Psychosocial Oncology
CFIR: Consolidated Framework for Implementation Research
CRF: Cancer-related fatigue
CS: Consumer/s
GP: General practitioner
HP: Health professional
NCCN: National Comprehensive Cancer Network
OT: Occupational therapist
PCFA: Prostate Cancer Foundation of Australia
