# Supplementary material for: Cancer related fatigue: implementing guidelines for optimal management
Source: BMC Health Serv Res. 2017 Jul 18;17:496. doi: 10.1186/s12913-017-2415-9 (PMC5516360; doi:10.1186/s12913-017-2415-9)
Supplement: Supplementary file 1 — Survey questionnaires HP1, C1, C2 and HP2. (DOCX 311 kb) [file 12913_2017_2415_MOESM1_ESM.docx]

Introduction to Study and Round 1 (both HP1 and C1)

This research project aims to identify issues with implementing a guideline for cancer-related fatigue (CRF) in Australia, and establish recommendations for practical application of the guideline including local adaptations. Thank you for contributing to this important research.

Cancer-related fatigue is a common symptom experienced by many people diagnosed with cancer [1]. Fatigue may be present before diagnosis of cancer and it is also a common side effect of radiotherapy and chemotherapy [2]. While CRF is often acute or short term, some people experience persistent CRF for months and years following treatment [3]. At moderate to high levels, CRF can affect physical and psychosocial quality of life including work and leisure [4]. The National Comprehensive Cancer Network (NCCN), an alliance of 23 world-leading US cancer centres, defined CRF:

*‘Cancer-related fatigue is a distressing persistent, subjective sense of physical, emotional and/or cognitive tiredness or exhaustion related to cancer or cancer treatment that is not proportional to recent activity and interferes with usual functioning*’ [1].

The method to be used in this research is called the Delphi technique. It has been used in many fields including health to establish expert consensus opinions [5]. Delphi participants are invited because they have expertise in the topic under investigation [6]. Advantages of the Delphi process are that participants in distant geographic locations can be included using (electronic) mail, and individual responses remain anonymous within the group [6].

Delphi involves several rounds of questionnaires completed by the same participants. Questions in later rounds are accompanied by a summary of results of the previous round including comments. New information may enable participants to modify their perceptions and each round builds upon the previous one.

This study will have two survey rounds that may be followed by telephone or email contact to clarify or expand upon answers. Individuals with specialist knowledge or experience of CRF have been invited to participate in this study. Participants are either cancer survivors who have experienced CRF or health professionals who are currently practising and are familiar with CRF and its impact. These include nurses, doctors, occupational therapists, physiotherapists, clinical psychologists, dieticians, social workers and health service managers. A broad representation across Australian states and territories and in different practice settings is expected.

The focus of Delphi 1 is to understand how participants view the acceptability, appropriateness and feasibility of the Pan-Canadian Guideline for Screening, Assessment and Management of Fatigue in Adults with Cancer-related Fatigue [7].

Please allow time to read the accompanying guideline before completing the questions in sections B and C. We ask you to record start, end of section and finish times because we want to know how long it takes people to answer the surveys.

- References

1. NCCN. *Cancer-related fatigue version 2.2015*. National Comprehensive Cancer Network (NCCN) Clinical Practice Guidelines in Oncology [Clinical guideline] 2015 16/7/2015]; Available from: <http://www.nccn.org/professionals/physician_gls/pdf/fatigue.pdf>.

2. Barsevick, A.M., et al., *Recommendations for high-priority research on cancer-related fatigue in children and adults.* Journal of the National Cancer Institute, 2013. 105(19): p. 1432-1440.

3. Bower, J.E., et al., *Screening, assessment, and management of fatigue in adult survivors of cancer: An American Society of Clinical Oncology clinical practice guideline adaptation.* Journal of Clinical Oncology, 2014. 32(17): p. 1840-50.

4. Minton, O., et al., *Cancer-related fatigue and its impact on functioning.* Cancer, 2013. 119(11 suppl): p. 2124-2130.

5. Sackman, H., *Delphi critique - expert opinion, forecasting and group process*, ed. T.R. Corporation. 1975, USA, Canada, England: Lexington Books.

6. Keeney, S., H. McKenna, and F. Hasson, *The Delphi Technique in Nursing and Health Research*. 2010, Wiley: Hoboken. p. 210.

7. Howell, D., et al., *A pan-Canadian practice guideline and algorithm: screening, assessment, and supportive care of adults with cancer-related fatigue.* Current Oncology, 2013. 20(3): p. e233-246.

HP1 Survey questions for Practitioners / Managers (Round 1)

Please enter your study ID (see registration form)

Please record the time you commenced this survey

1. This section is about your past and current experience of clinical guidelines for CRF.
   1. Do you consider there is a need for clinical guidelines for management of CRF? (Yes / No / Unsure)
   2. Have you used or a CRF guideline as a practitioner? Yes / No / Uncertain
   3. (If yes) Which guidelines did you use? (Free text)
   4. Did you find these guidelines were easily implemented? Yes / No + comment

If you work in more than one health care service please answer the next four questions about one practice location.

- 1. Practice type – please choose the type that best describes your health care facility (from drop down box options)

|  | General hospital |
| --- | --- |
|  | Cancer centre |
|  | General practice |
|  | Private hospital |
|  | Private practice (specialty) |
|  | Rehabilitation |
|  | Community health |
|  | Domiciliary service |
|  | Inpatient hospice |
|  | Community palliative care |
|  | Other (free text) |

- 1. How is fatigue currently assessed at your workplace? Which practitioners are responsible for each (if known)? (Free text)

| Assessment method | Assessments used | Practitioner discipline | |
| --- | --- | --- | --- |
|  |  | Actual | Potential |
| *Example*  *Screening for fatigue* | *Supportive needs screening tool* | *Nurse Coordinator* | *Allied Health Assistant* |
| Screening for fatigue  e.g. 0-10 scale |  |  |  |
| History and impact of CRF |  |  |  |
| Risk factors for fatigue |  |  |  |
| Physical exam |  |  |  |
| Symptom review |  |  |  |
| Laboratory tests |  |  |  |

- 1. Which main elements of fatigue guidelines are currently implemented in your workplace or treatment centre? (One answer per line)

| Main guideline element | Implemented | | | | |
| --- | --- | --- | --- | --- | --- |
|  | Always | Often | Occasion-ally | Never | Unsure |
| Screening for fatigue at routine time points – e.g. at start of treatment or new treatment cycle, during or at end of treatment |  |  |  |  |  |
| Routine education about fatigue and counselling about management |  |  |  |  |  |
| Comprehensive Assessment: History, Laboratory tests and physical exam (for moderate to severe fatigue) |  |  |  |  |  |
| Treatment of contributing factors (moderate to severe fatigue) |  |  |  |  |  |
| Non-pharmacological interventions for moderate to severe fatigue described in p26-28 of the guideline – eg exercise, psychosocial interventions |  |  |  |  |  |

- 1. Please indicate your level of agreement with the following statement:

|  | Strongly disagree | Disagree | Neutral | Agree | Strongly agree |
| --- | --- | --- | --- | --- | --- |
| I am satisfied with current approaches to CRF management at my workplace (1) |  |  |  |  |  |

Would you like to add answers for another workplace? (Yes - Repeat the above)

B. This section examines the practical aspects of implementing the pan-Canadian guideline

| Please indicate your level of agreement with the following statements about recommendations for screening and assessment of fatigue in the pan-Canadian guideline: | | | | | | | | | | | | | |
| --- | --- | --- | --- | --- | --- | --- | --- | --- | --- | --- | --- | --- | --- |
|  | Strongly disagree | | Disagree | | | Neutral | | | Agree | | | Strongly agree | |
| 1. The guideline contains sufficient detail to implement: Screening for fatigue |  | |  | | |  | | |  | | |  | |
| 1. The guideline contains sufficient detail to implement: Focused fatigue assessment |  | |  | | |  | | |  | | |  | |
| 1. The guideline contains sufficient detail to implement: Measurement of fatigue |  | |  | | |  | | |  | | |  | |
| 1. In what ways could the guidelines about screening and assessing fatigue be enhanced for implementation? | (Free text) | | | | | | | | | | | | |
| Please indicate your level of agreement with the following statements about recommendations for assessing contributing factors in the pan-Canadian CRF guideline: | | | | | | | | | | | | | |
|  | | Strongly disagree | | Disagree | | | Neutral | | | Agree | | | Strongly agree |
| 1. The guideline contains sufficient detail to assess: Treatment complications | |  | |  | | |  | | |  | | |  |
| 1. The guideline contains sufficient detail to assess:   Weight/caloric intake changes | |  | |  | | |  | | |  | | |  |
| 1. The guideline contains sufficient detail to assess:   Fluid / electrolyte balance | |  | |  | | |  | | |  | | |  |
| 1. The guideline contains sufficient detail to assess:   Medications | |  | |  | | |  | | |  | | |  |
| (Continued) Please indicate your level of agreement with the following statements about recommendations for assessing contributing factors in the pan-Canadian CRF guideline: | | | | | | | | | | | | | |
| 1. The guideline contains sufficient detail to assess:   Other symptoms / side effects | |  | |  | | |  | | |  | | |  |
| 1. The guideline contains sufficient detail to assess:   Activity level changes | |  | |  | | |  | | |  | | |  |
| 1. The guideline contains sufficient detail to assess:   Co-morbid conditions | |  | |  | | |  | | |  | | |  |
| 1. How could the guidelines about assessing contributing factors be enhanced for implementation? | | (Free text) | | | | | | | | | | | |
| Physical examination | | Strongly disagree | | | Disagree | | | Neutral | | | Agree | | Strongly agree |
| 1. The guideline contains sufficient detail to assess:   Gait, posture, range of motion, muscle wasting | |  | | |  | | |  | | |  | |  |
| 1. The guideline contains sufficient detail to assess:   Eyes, oral assessment | |  | | |  | | |  | | |  | |  |
| 1. The guideline contains sufficient detail to assess:   Tachycardia, shortness of breath | |  | | |  | | |  | | |  | |  |
| 1. How could the guidelines about physical examination be enhanced for implementation? | | (Free text) | | | | | | | | | | | |

| Please indicate your level of agreement with the following statements about recommendations for treatments in the pan-Canadian CRF guideline: | | | | | |
| --- | --- | --- | --- | --- | --- |
|  | Strongly disagree | Disagree | Neutral | Agree | Strongly agree |
| 1. The guideline contains sufficient detail to provide Education and counselling |  |  |  |  |  |
| 1. The guideline contains sufficient detail to prescribe or provide non-pharmacological interventions for moderate and severe fatigue |  |  |  |  |  |
| 1. How could the guidelines about education and interventions for fatigue be enhanced for implementation? | (Free text) | | | | |
| 1. Which elements are impractical to implement? Please explain why | (Free text) | | | | |
| 1. What adaptations to the guideline are needed to enable it to be trialled or implemented in your workplace? | (Free text) | | | | |

C. These questions are about your perceptions about the Pan-Canadian fatigue guideline.

Top of Form

| Please indicate your level of agreement with the following statements: | | | | | |
| --- | --- | --- | --- | --- | --- |
|  | Strongly disagree | Disagree | Neutral / unsure | Agree | Strongly agree |
| 1. The benefits of the pan-Canadian guideline outweigh the costs, inconvenience or discomfort |  |  |  |  |  |
| 1. The pan-Canadian CRF guideline recommendations are consistent with existing attitudes and practices in my workplace |  |  |  |  |  |
| 1. The recommendations of the pan-Canadian CRF guideline could be carried out with minimal additional training |  |  |  |  |  |
| 1. In its current form, would you adopt, reject or trial the pan-Canadian CRF guideline? | Reject | Delay decision | Trial | Adopt | Please state why (optional free text) |

| 1. What are the five most important factors that would encourage you to adopt the pan-Canadian CRF guideline? (Multi choice, check five) | |
| --- | --- |
| 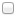 | If service leaders, management or government endorsed the guideline |
| 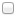 | If use were mandated by health service with performance indicators (audit) |
| 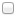 | Favourable opinions of colleagues or services who use the guideline |
| 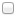 | If it is compatible with current practices |
| 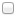 | If the procedures are easily put into practice |
| 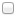 | If forms, resources and referral pathways are available electronically |
| 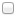 | If I could have an opportunity to trial the guideline before deciding |
| 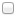 | If practitioners could choose when/not to use it |
| 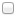 | If financial or other incentives to use the guideline were offered |
| 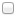 | Availability of published evidence of the guideline’s effectiveness |
| 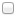 | Patient requests for fatigue assessment and management |
| 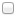 | Additional funding provided for staff to implement the guideline or other care |
| 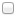 | Availability of staff educational materials or additional training in the guideline procedures |
| 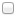 | If decision support tools and reminders were available |
| 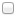 | Other (explain) |

Bottom of Form

Please record the time you finished the survey

Thank you for participating in Round 1. Your input is invaluable and we hope that you will support this project in the second survey round.

The research team will now prepare a summary of the group results. This may take several weeks. You will have an opportunity to reconsider your answers after reading the summary and change them in the next round if you wish. In Round 2 there may be new questions arising from the results of Round 1.

- END OF HP1 -

C1 Survey questions for Consumers (Round 1)

Please enter your study ID (see registration form)

Please record the time you commenced this survey

A. This section is about your past and current experience of clinical guidelines for CRF.

If you attended more than one health care facility for cancer care please answer the following questions about one facility at a time. You will be able to answer for up to three facilities if you wish.

1. What category best describes the health care facility or service you attended for cancer care?

This means the type of organisation where you had your cancer treatment or follow up.
Please check one box only.

Top of Form

⬜ Public hospital - general

⬜ Specialist cancer centre

⬜ Private hospital – general

⬜ Private hospital - oncology

⬜ General practice / primary health care

⬜ Specialist private practice

⬜ Rehabilitation eg physio or program

⬜ Community health

⬜ Palliative care

⬜ Inpatient hospice

⬜ Domiciliary service
(care or treatment in your home)

⬜ Other (please describe) ____________________________

2. What types of health care or treatment have you received at this facility?

e.g. tests, medical care, treatment, physio etc

3. Please indicate your level of agreement with the following statement:

I am satisfied with current approaches to CRF management at my health provider (1)

| Strongly disagree | Disagree | Neutral | Agree | Strongly agree |
| --- | --- | --- | --- | --- |
| ⬜ | ⬜ | ⬜ | ⬜ | ⬜ |

4. Do you consider there is a need for clinical guidelines for management of CRF tailored for the Australian context?

| Yes | Maybe | No |
| --- | --- | --- |
| ⬜ | ⬜ | ⬜ |

5. Are you aware of any CRF guideline used by any of the health professionals at this health care facility?

| Yes | Uncertain | No |
| --- | --- | --- |
| ⬜ | ⬜ | ⬜ |

6. How often have you experienced the following fatigue assessments at your health care provider and which practitioners conducted the assessments?

The example shows you have had an assessment once by a nurse

|  | How frequently were you assessed? | | | |  |
| --- | --- | --- | --- | --- | --- |
|  | Often | A few times | Once | Never | Health care worker |
| *Example assessment* |  |  | ✔ |  | *Nurse* |
| A health worker asked you if you had fatigue at defined times  e.g. start of treatment or cycle, end of treatment |  |  |  |  |  |
| Someone asked you about your experience of CRF e.g. when it started, how it feels and affects your life |  |  |  |  |  |
| You were asked about risk factors for fatigue  e.g. sleep, medications, other conditions |  |  |  |  |  |
| You had a physical examination  e.g. muscle test, eyes, pulse, walk test |  |  |  |  |  |
| Your practitioner checked for symptoms associated with fatigue (pain, anxiety, depression, breathlessness) |  |  |  |  |  |
| You had blood tests or scans to see whether an infection or other condition might be causing fatigue |  |  |  |  |  |

7. Would you like to add answers for another health care facility you have attended?

⬜ Yes ⬜ No – please skip to question 19.

8. What category best describes the second health care facility or service you attended for cancer care?

Please check one box only.

Top of Form

⬜ Public hospital - general

⬜ Specialist cancer centre

⬜ Private hospital – general

⬜ Private hospital - oncology

⬜ General practice / primary health care

⬜ Specialist private practice

⬜ Rehabilitation

⬜ Community health

⬜ Palliative care

⬜ Inpatient hospice

⬜ Domiciliary service
(care or treatment in your home)

⬜ Other (please describe) ____________________________

9. What types of health care or treatment have you received at this facility (2)?

e.g. tests, medical care, treatment, physio etc

10. Please indicate your level of agreement with the following statement:

I am satisfied with current approaches to CRF management at my health provider (2)

| Strongly disagree | Disagree | Neutral | Agree | Strongly agree |
| --- | --- | --- | --- | --- |
| ⬜ | ⬜ | ⬜ | ⬜ | ⬜ |

11. Are you aware of any CRF guideline used by any of the health professionals at this health care facility (2)?

| Yes | Uncertain | No |
| --- | --- | --- |
| ⬜ | ⬜ | ⬜ |

12. How often have you experienced the following fatigue assessments at your health care provider (2) and which practitioners conducted the assessments?

|  | How frequently were you assessed? | | | |  |
| --- | --- | --- | --- | --- | --- |
|  | Often | A few times | Once | Never | Health care worker |
| A health worker asked you if you had fatigue at defined times  e.g. start of treatment or cycle, end of treatment |  |  |  |  |  |
| Someone asked you about your experience of CRF e.g. when it started, how it feels and affects your life |  |  |  |  |  |
| You were asked about risk factors for fatigue  e.g. sleep, medications, other conditions |  |  |  |  |  |
| You had a physical examination  e.g. muscle test, eyes, pulse, walk test |  |  |  |  |  |
| Your practitioner checked for symptoms associated with fatigue (pain, anxiety, depression, breathlessness) |  |  |  |  |  |
| You had blood tests or scans to see whether an infection or other condition might be causing fatigue |  |  |  |  |  |

13. Would you like to add answers for a third health care facility you have attended?

⬜ Yes ⬜ No – please skip to question 19.

14. What category best describes the third health care facility or service you attended for cancer care?

Please check one box only.

Top of Form

⬜ Public hospital - general

⬜ Specialist cancer centre

⬜ Private hospital – general

⬜ Private hospital - oncology

⬜ General practice / primary health care

⬜ Specialist private practice

⬜ Rehabilitation

⬜ Community health

⬜ Palliative care

⬜ Inpatient hospice

⬜ Domiciliary service
(care or treatment in your home)

⬜ Other (please describe) ____________________________

15. What types of health care or treatment have you received at this facility (3)?

e.g. tests, medical care, treatment, physio etc

16. Please indicate your level of agreement with the following statement:

I am satisfied with current approaches to CRF management at my health provider (3)

| Strongly disagree | Disagree | Neutral | Agree | Strongly agree |
| --- | --- | --- | --- | --- |
| ⬜ | ⬜ | ⬜ | ⬜ | ⬜ |

17. Are you aware of any CRF guideline used by any of the health professionals at this health care facility (3)?

| Yes | Uncertain | No |
| --- | --- | --- |
| ⬜ | ⬜ | ⬜ |

18. How often have you experienced the following fatigue assessments at your health care provider (3) and which practitioners conducted the assessments?

|  | How frequently were you assessed? | | | |  |
| --- | --- | --- | --- | --- | --- |
|  | Often | A few times | Once | Never | Health care worker |
| A health worker asked you if you had fatigue at defined times  e.g. start of treatment or cycle, end of treatment |  |  |  |  |  |
| Someone asked you about your experience of CRF e.g. when it started, how it feels and affects your life |  |  |  |  |  |
| You were asked about risk factors for fatigue  e.g. sleep, medications, other conditions |  |  |  |  |  |
| You had a physical examination  e.g. muscle test, eyes, pulse, walk test |  |  |  |  |  |
| Your practitioner checked for symptoms associated with fatigue (pain, anxiety, depression, breathlessness) |  |  |  |  |  |
| You had blood tests or scans to see whether an infection or other condition might be causing fatigue |  |  |  |  |  |

19. End of section A.

Please write down the time you finished Section A ­________________

B. This section examines the acceptability of the pan-Canadian guideline to people with cancer-related fatigue. Please refer to pages in the guideline summary as indicated for each question.

Please write down the time you started Section B ­________________

Please indicate your level of agreement with the following statements about recommendations for screening and assessment of fatigue in the pan-Canadian guideline:

|  | Strongly disagree | Disagree | Neutral | Agree | Strongly agree |
| --- | --- | --- | --- | --- | --- |
| 20. Recommendations for screening for fatigue are acceptable to me (p6 of 14) | ⬜ | ⬜ | ⬜ | ⬜ | ⬜ |
| 21. Recommendations for focused fatigue assessment are acceptable to me (p6-7) | ⬜ | ⬜ | ⬜ | ⬜ | ⬜ |
| 22. Recommendations for measurement of fatigue are acceptable to me (p6-7) | ⬜ | ⬜ | ⬜ | ⬜ | ⬜ |

23. How easy do you think the assessment process would be for a consumer with fatigue?

| Very difficult | Difficult | Neutral | Easy | Very easy |
| --- | --- | --- | --- | --- |
| ⬜ | ⬜ | ⬜ | ⬜ | ⬜ |

Please explain your answer.

24. In what ways could the recommendations for *screening and assessing fatigue* be made more acceptable to consumers?

25. From your perspective, when would be the best time points to have fatigue screening?

Please indicate your level of agreement with the following statements about treatment recommendations in the pan-Canadian CRF guideline:

|  | Strongly disagree | Disagree | Neutral | Agree | Strongly agree |
| --- | --- | --- | --- | --- | --- |
| 26. The recommendations about Education and counselling are clear and acceptable to me (p10-11) | ⬜ | ⬜ | ⬜ | ⬜ | ⬜ |
| 27. The recommendations about Non-pharmacological interventions for moderate and severe fatigue are clear and acceptable to me (p9-11) | ⬜ | ⬜ | ⬜ | ⬜ | ⬜ |

28. How could the guidelines for *education and interventions for fatigue* be made more acceptable to consumers (i.e. fit with what consumers are likely to want or expect)?

29. What is needed to make the guideline feasible (i.e. easy to use) from your perspective as a consumer?

30. From your perspective, when would be a useful time to receive advice for managing or preventing fatigue?

31. What is your preferred format/s to receive educational material about fatigue? If you choose more than one, please add a comment to explain why.

⬜ Leaflet / printed material

⬜ DVD

⬜ Group education session/s

⬜ Individual counselling

⬜ Web page information

⬜ On-line interactive

⬜ Other (specify) ________________

Comments

________________________________________________________________________________

________________________________________________________________________________

Going well - you’re almost at the end of the survey!C. The next questions are about your perceptions about the Pan-Canadian fatigue guideline.

Please indicate your level of agreement with the following statements:

|  | Strongly disagree | Disagree | Neutral / unsure | Agree | Strongly agree |
| --- | --- | --- | --- | --- | --- |
| 32. The benefits of the pan-Canadian guideline outweigh any costs, inconvenience or discomfort to me | ⬜ | ⬜ | ⬜ | ⬜ | ⬜ |
| 33. The pan-Canadian guideline recommendations match with my expectations | ⬜ | ⬜ | ⬜ | ⬜ | ⬜ |

34. What are the *five* most important factors that would influence you to use the pan-Canadian CRF guideline? Please check only five.

| ⬜ If clear written or on-line materials were available for consumers |
| --- |
| ⬜ If my health professional promoted its use |
| ⬜ If it were endorsed by a leading Australian cancer organisation |
| ⬜ If other people with CRF recommend it personally or in testimonials |
| ⬜ Availability of health professionals with expertise to assess and treat CRF |
| ⬜ If fatigue is taken seriously by my health care providers |
| ⬜ If I could attend a ‘one-stop shop’ for fatigue |
| ⬜ The waiting list for assessment or treatments is not too long |
| ⬜ If there are minimal costs involved to use |
| ⬜ If there were proof that it works |
| ⬜ Convenient service location |
| ⬜ If a self-assessment component that I could do at home were available |
| ⬜ Support from family and friends to enable participation in assessment or programs |
| ⬜ Other (explain)  __________________________________________________________________ |

You have now reached the end of survey!

Please write down the time you finished. ­­­­­­­­­_____________________

Thank you for participating in Round 1. Your input is invaluable and we look forward to your support in the second survey round.

The research team will now prepare a summary of the group results. This may take several weeks. You will have an opportunity to review your answers after reading the summary. Round 2 will contain new questions to further analyse results from Round 1. We expect to be able to run Round 2 in mid-February 2016. We want to enable your further participation so please advise the study team if this time frame is difficult for you. We will do our best to make special provisions.

Once again, thanks for your involvement so far.

With kind regards from the research team.

Please return the completed survey to:

Name,
(Mailing address provided)

- END OF C1 -

C2 Survey questions for consumers (Round 2)

Please enter your study ID (see registration form)

Please record the time you commenced this survey

Welcome to Survey 2. The questions in this survey have arisen from results, comments and suggestions by consumers in Survey 1. Please read and refer to the summary of results provided as needed.

Presence of fatigue

Screening for fatigue involves one or two questions and takes about 2 minutes. Recommendations for fatigue screening in the Canadian guideline were acceptable to 94% of consumers who answered this question.

The Canadian guideline does not recommend specific time-points for fatigue screening. Study participants listed many time-points as important for fatigue screening.

Thinking of your own fatigue experience, how important would each point of time on this schedule be to you?

| Fatigue SCREENING time point | Very important | Important | Optional | Not needed |
| --- | --- | --- | --- | --- |
| 1. At diagnosis or start of treatment to compare to later | ⬜ | ⬜ | ⬜ | ⬜ |
| 1. During routine assessment before each new treatment, chemotherapy cycle or radiotherapy course | ⬜ | ⬜ | ⬜ | ⬜ |
| 1. At the end of a treatment course | ⬜ | ⬜ | ⬜ | ⬜ |
| 1. At one month after end of treatment | ⬜ | ⬜ | ⬜ | ⬜ |

Thinking of your own fatigue experience, how important would each fatigue screening point of time on this schedule be to you?

| Fatigue SCREENING time point | Very important | Important | Optional | Not needed |
| --- | --- | --- | --- | --- |
| 1. At 3 months after end of treatment | ⬜ | ⬜ | ⬜ | ⬜ |
| 1. At 6 months after end of treatment | ⬜ | ⬜ | ⬜ | ⬜ |
| 1. At yearly check-ups | ⬜ | ⬜ | ⬜ | ⬜ |
| 1. After hospitalisation or changed health status | ⬜ | ⬜ | ⬜ | ⬜ |

1. Which of the following approaches to fatigue screening would be acceptable to you? You may choose as many as you like.

- Ask me about my fatigue level during routine appointments
- Ask me about my fatigue level when asking about pain, nausea and other symptoms
- Tell me about the possibility of fatigue at diagnosis and show me how to monitor it myself, and inform my health professionals when it is a problem
- Give me a short questionnaire to fill in while in waiting room, to discuss with health professional(s) at that visit
- Give me access to a mobile phone app for rating my symptoms including fatigue when I want so I can tell my health professional when it is a problem
- Other (comment)

Please indicate your level of agreement with the following three statements:

1. It is essential to be made aware of the possibility of fatigue and how to measure it, when you are first diagnosed with cancer.

| Strongly disagree | Disagree | Neutral | Agree | Strongly agree |
| --- | --- | --- | --- | --- |
| ⬜ | ⬜ | ⬜ | ⬜ | ⬜ |

1. More detailed information about fatigue prevention can wait until treatment has started.

| Strongly disagree | Disagree | Neutral | Agree | Strongly agree |
| --- | --- | --- | --- | --- |
| ⬜ | ⬜ | ⬜ | ⬜ | ⬜ |

1. A screening hand out could contain simple information about how to rate your fatigue level, and what to do for different levels of fatigue.
   e.g.

| Strongly disagree | Disagree | Neutral | Agree | Strongly agree |
| --- | --- | --- | --- | --- |
| ⬜ | ⬜ | ⬜ | ⬜ | ⬜ |

12a. Please comments or suggestions about the flow-chart if you wish (Optional)

Assessing fatigue

Fatigue levels usually change over the day or week, therefore the answers to one or two screening questions do not tell the full story. If fatigue is rated more than 3/10, longer questionnaires are needed to accurately measure fatigue to enable changes to be identified, as well as factors that might be contributing to fatigue. This should be followed by a discussion with a health professional. Survey participants called for a flexible, individual approach to fatigue management.

1. How would you prefer to fill in a longer questionnaire? [Choose one or more]

- At home before an appointment
- By phone
- In a waiting room
- During an appointment
- Whenever I want to
- None of the above (please comment)

Please indicate your level of agreement with the following statement:

| 1. I would like to be given the choice of doing a paper, electronic or verbal questionnaire to assess my fatigue | | | | |
| --- | --- | --- | --- | --- |
| Strongly disagree | Disagree | Neutral | Agree | Strongly agree |
| ⬜ | ⬜ | ⬜ | ⬜ | ⬜ |
| 1. What mode of questionnaire would you prefer to assess your fatigue? | | | | |
| Paper and pen | Electronic | Verbal / discussion | No preference | Other *Please explain* |
| ⬜ | ⬜ | ⬜ | ⬜ |  |

| Please indicate your level of agreement with the following statements: | | | | |
| --- | --- | --- | --- | --- |
| 1. It is important to me to have some say in when, where and how I am assessed if I have moderate to severe fatigue | | | | |
| Strongly disagree | Disagree | Neutral | Agree | Strongly agree |
| ⬜ | ⬜ | ⬜ | ⬜ | ⬜ |
| 1. If I have fatigue I need health professionals to be caring and to consider my side effects such as nausea, chemo-brain and physical limitations. | | | | |
| Strongly disagree | Disagree | Neutral | Agree | Strongly agree |
| ⬜ | ⬜ | ⬜ | ⬜ | ⬜ |
| 1. I would prefer a longer appointment compared to extra visits. | | | | |
| Strongly disagree | Disagree | Neutral | Agree | Strongly agree |
| ⬜ | ⬜ | ⬜ | ⬜ | ⬜ |

Education and resources.

Many participants (63%) wanted some advice for managing or preventing fatigue at or soon after diagnosis. However, several noted that the amount of information given at that time could be overwhelming. It is known that at times of stress, people typically struggle to remember new information^^[[1]](#footnote--1)^^.

Please indicate your level of agreement with the following statements:

| 1. Information and education about fatigue should be offered at different levels of detail (e.g. basic, standard, detailed) | | | | |
| --- | --- | --- | --- | --- |
| Strongly disagree | Disagree | Neutral | Agree | Strongly agree |
| ⬜ | ⬜ | ⬜ | ⬜ | ⬜ |
| 1. Access to individual or group education about fatigue supported by written material is important to me | | | | |
| Strongly disagree | Disagree | Neutral | Agree | Strongly agree |
| ⬜ | ⬜ | ⬜ | ⬜ | ⬜ |
| 1. Information about how to get help for fatigue management in my local area would be useful e.g. education, counselling, meditation, physical training, support groups | | | | |
| Strongly disagree | Disagree | Neutral | Agree | Strongly agree |
| ⬜ | ⬜ | ⬜ | ⬜ | ⬜ |

D. Service provision

Several participants reported that a change to person-centred care is needed. The next questions examine how a person-centred model of care might be put into practice.

Please indicate your level of agreement with the following statements:

| 1. Fatigue management should be a part of routine cancer services | | | | |
| --- | --- | --- | --- | --- |
| Strongly disagree | Disagree | Neutral | Agree | Strongly agree |
| ⬜ | ⬜ | ⬜ | ⬜ | ⬜ |
| 1. I want to know which health professional is overseeing and monitoring my fatigue levels and supporting me | | | | |
| Strongly disagree | Disagree | Neutral | Agree | Strongly agree |
| ⬜ | ⬜ | ⬜ | ⬜ | ⬜ |
| 1. Automatic bookings for non-essential appointments such as education or exercise might help me attend, especially if I am feeling tired. | | | | |
| Strongly disagree | Disagree | Neutral | Agree | Strongly agree |
| ⬜ | ⬜ | ⬜ | ⬜ | ⬜ |

Some participants told us to include real comments about the physical and emotional impacts of fatigue in education for professionals

Please indicate your level of agreement with the following statements:

| 25. On-line education about managing fatigue should be available to all health professionals involved in my cancer care | | | | |
| --- | --- | --- | --- | --- |
| Strongly disagree | Disagree | Neutral | Agree | Strongly agree |
| ⬜ | ⬜ | ⬜ | ⬜ | ⬜ |
| 26. Make it real. I would like health professionals to know about how fatigue has affected real people like me. | | | | |
| Strongly disagree | Disagree | Neutral | Agree | Strongly agree |
| ⬜ | ⬜ | ⬜ | ⬜ | ⬜ |

The guideline layout and language

Some participants commented that the language used in the guideline should be clear and simple without jargon. Participants suggested a range of formats including pamphlets, workshops and websites.

Please indicate your level of agreement with the following statements:

| 27. I find diagrams such as flow-charts helpful to understand and learn new information | | | | |
| --- | --- | --- | --- | --- |
| Strongly disagree | Disagree | Neutral | Agree | Strongly agree |
| ⬜ | ⬜ | ⬜ | ⬜ | ⬜ |
| 28. The guideline should be written for consumers in lay language with a simple rationale for each recommendation e.g. why walking would be of benefit | | | | |
| Strongly disagree | Disagree | Neutral | Agree | Strongly agree |
| ⬜ | ⬜ | ⬜ | ⬜ | ⬜ |

29. Any final thoughts about using the Canadian Guidelines for cancer-related fatigue in Australia taking into consideration your location or diagnosis? (Optional) (Free text)

You have reached the end of the survey!

Your time and effort in participating in this research is greatly appreciated. Understanding the opinions of Australians who have experienced cancer-related fatigue is essential for planning how to put the Canadian CRF guidelines into practice. Results from this study, together with the parallel health professional study results, will be used to develop recommendations for a trial of the guidelines. You will receive a summary of the key findings and recommendations by the end of July 2016.

Feedback on your experience of participating in this project is most welcome. Please contact Name at (email address).

With many thanks and best wishes from the research team – and the study reference panel.

Please return your survey in the reply-paid envelope to Name

(Lead researcher – mailing address provided)

- END OF C2 -

HP2 Survey questions for Practitioners / Managers (Round 2)

*Note to Ethics: This will be presented as an electronic survey using Qualtrics therefore has not been formatted for hard copy. Possible responses are in parentheses.
(Agree/disagree refers to 5-point Likert scaling of strongly agree to strongly disagree.)*

Instructions to participants:

The questions in this survey have arisen from results, comments and suggestions by health professionals in Survey 1. Please read and refer to the summary of results provided. If you would like additional detail, please request a copy of the detailed result by email to Name (lead researcher, email provided).

Screening for presence of fatigue.

74% of respondents agreed that the guideline contained sufficient detail to implement screening for fatigue, with 10% disagreeing and 16% neutral.

1. Should fatigue screening be included in a multi-symptom checklist or a fatigue-specific tool? (Multi / fatigue only / both / unsure)
2. Would a short-list of accessible valid fatigue screening tools be helpful? (Yes/no/unsure) e.g.
   - Single-item “How would you rate your fatigue on a scale of 0 to 10 with 0 being ‘no fatigue’ and 10 being the ‘worst possible fatigue’?”
   - Tiredness item from ESAS^^[[2]](#footnote-0)^^ for advanced cancer or SCNST^1^ for general use
   - Fatigue Pictogram - 2-items, past week
3. Which are essential time points for fatigue screening in Australia? (Please mark those you consider should be minimum standard)
   (At diagnosis / start of treatment cycle / each review / end of treatment / 3 months post / 6 months post / annual / if previously screened positive / change in condition / changed phase of care)

Please indicate your level of agreement with the statements (agree/disagree)

1. ‘All clinicians should be able to screen for presence of fatigue’
2. ‘Once clinicians identify moderate to severe fatigue they should seek advice and/or refer for comprehensive assessment’

Focused fatigue assessment

76% of participants agreed that the guideline contained sufficient detail to conduct a focused fatigue assessment, while 13% disagreed and 10% could not decide.

1. ‘A self-report questionnaire (such as the Brief Fatigue Inventory) that measures fatigue severity and impact could be useful as part of focused fatigue assessment and history taking’ (agree / disagree)
2. ‘Applying standardised diagnostic criteria for CRF is useful in the clinical setting to distinguish CRF from other types of fatigue’ (agree/disagree)

Fatigue measurement

68% of participants agreed that there was sufficient detail in the guideline to measure fatigue, 13% disagreed and 13% were neutral.

1. ‘A recommended valid patient self-report measure for fatigue and related constructs would be helpful to monitor progress across settings’ (agree/disagree)

Comprehensive assessment of fatigue

50% of health professionals agreed that there was sufficient detail to implement most of the elements for the comprehensive fatigue assessment, while 28% disagreed and the remainder were undecided

1. ‘An electronic checklist of contributing/risk factors for fatigue, with space to enter which assessments were performed, would be a useful clinical tool’ (agree/ disagree)
2. ‘A self-assessment for patient to identify issues would be time-efficient for clinicians’ (agree/disagree)
3. ‘The guideline should contain links to additional detail about specific contributing factors *such as* medications, electrolyte levels, nutritional parameters and rate of physical activity change’ (agree/disagree)
4. Would a stratified approach to comprehensive assessment of CRF be clinically feasible? One example –
   Step 1 – Focused fatigue assessment
   Step 2 – Self-assessment tool/s, vital signs, other symptoms, weight
   Step 3 – Further assessment of high risk items on tool eg medications, comorbidities
   Step 4 – Blood/pathology tests or scans as relevant
   Step 5 - Physical examination – muscle bulk, gait / transfers / endurance
   (Yes/no/unsure)
5. Please add your suggestions about stratification of fatigue assessment including comment on the order in the example above (free text, optional)

Physical examination

37% of respondents agreed that the guidelines contained sufficient detail to implement physical assessment. 39% disagreed and the remainder were undecided.

1. ‘The guideline should contain links to additional detail about physical examinations and significance of findings’ (agree/disagree)
2. ‘A practice nurse (or other designated professional) could screen for tachycardia, shortness of breath and signs of nutritional deficiencies (oral) and anaemia (eyes) and refer to the appropriate professional for further assessment’ (agree/disagree)
3. ‘Gait, posture, muscle wasting and range of motion would ideally be assessed by a relevant health professional – if appropriate’ (agree/disagree)

Algorithm for CRF

Some participants considered the fatigue management algorithm is a useful summary of the guideline although lacking in detail, particularly for those unfamiliar with the field. Some respondents thought the algorithm was ambiguous, as it appears to be a checklist without spaces to record tests performed and results. Participants suggested several enhancements.

1. Please indicate which enhancements you would use (checklist, multiple answers)

- Electronic format
- Hyperlinks to details of assessments, interventions and referrals
- *Appendices* with details of assessments and interventions
- Links to (electronic) checklists and forms with prompts and text entry capacity
- A mobile application
- Wiki system
- Stratified assessment guide (i.e. break it into stages - what to do first)
- Other

Clinical resources

Several respondents observed that clinical resources are already limited and guideline implementation should be consistent with existing practice and frameworks.
- Clarification of which professionals were qualified / responsible for which elements of fatigue screening and assessment was considered helpful by some participants.
- A need to develop referral pathways and links to providers of intervention services was identified.
- The use of evidence-based self-management handouts or web-based content such as [Dr Mike Evans on Fatigue](https://www.youtube.com/watch?v=YTFPMYGe86s) YouTube clip was suggested, to replace or reinforce verbal education.

- 1. Which self-management support handout/s or web sites (if any) do you recommend to support verbal information? (free text)
  2. ‘Referral pathways with capacity for local adaptation would be clinically useful’ (agree/disagree)
  3. ‘Determination of which health professionals should take responsibility for assessments, interventions and follow up would improve consistency of practice’ (agree/disagree)
  4. What other decision support tools would be useful in your clinical setting? (free text)

Education for health professionals about fatigue management

Factors influencing the success of guideline implementation include effective education and promotion strategies^[[3]](#footnote-1)^.
Increasing health professionals’ awareness of CRF management through targeted education and engagement of general practitioners were each mentioned by several participants.
A number of participants identified the need for multidisciplinary teamwork in managing CRF.

1. Would you use a guide to communicating with patients about fatigue e.g. ‘How to discuss fatigue’? (Yes/no unsure)
2. What is your preferred method/s of learning about assessment and management of CRF? (checklist, multiple answers)

- Power point slides or static web page
- Workshop / seminar at my workplace
- Workshop / seminar at conference or meeting
- Workshop / seminar via distance education e.g. Videoconference, Interactive website, Webinar
- Other (please comment) ­­­__­­________

1. What type of content is preferred? (checklist, single answer)

- Specific education e.g. how to assess or deliver non-pharmacological interventions such as education, CBT and exercise
- Generic health professional education about using the guideline
- Both of the above
- Neither of the above
- Other (please comment) ­­­__­­________

1. Do you have any further comments about implementing guidelines for cancer-related fatigue in Australia? (eg related to your practice type) (Free text)

You have reached the end of the survey. Please click the button below to exit the survey.

Final text for participants:

Many thanks for your time and effort in participating in this research, it is greatly appreciated. Cancer-related fatigue is a multidisciplinary issue and your perspective broadens the generalisability of results to beyond a single discipline, practice location or type. Results will be used to inform recommendations for an implementation study. You will receive a summary of the key findings and recommendations in due course.

Best wishes from the research team – and the study reference panel.

- END OF HP2 -

1. Schwabe L, Joels M, Roozendaal B, Wolf OT, Oitzl MS (2011) Stress effects on memory: an update and integration. Neurosci Biobehav Rev 36: 1740-1749 doi:10.1016/j.neubiorev.2011.07.002 [↑](#footnote-ref--1)
2. These abbreviations will be familiar to the participants [↑](#footnote-ref-0)
3. Prior M, Guerin M, Grimmer‐Somers K (2008) doi:10.1111/j.1365-2753.2008.01014.x [↑](#footnote-ref-1)
