# Supplementary material for: Cancer related fatigue: implementing guidelines for optimal management
Source: BMC Health Serv Res. 2017 Jul 18;17:496. doi: 10.1186/s12913-017-2415-9 (PMC5516360; doi:10.1186/s12913-017-2415-9)
Supplement: Supplementary file 2 — Results of all quantitative survey questions. (DOCX 119 kb) [file 12913_2017_2415_MOESM2_ESM.docx]

**RESULTS: Theme 1 - Guideline content or layout**

(Consensus statements are bolded)

| Domain | Enhancement or statement | Survey | N | Agree (n) | Agree (%) |
| --- | --- | --- | --- | --- | --- |
| Presentation: guideline enhancements you would use | Appendices with details of assessments / interventions | HP2 | 31 | 25 | 80.6 |
|  | Stratified assessment guide | HP2 | 31 | 24 | 77.4 |
|  | Electronic format | HP2 | 31 | 22 | 71.0 |
|  | Hyperlinks to details of assessments / interventions / forms | HP2 | 31 | 20 | 64.5 |
|  | Links to checklists and forms with prompts and text entry capacity | HP2 | 31 | 18 | 58.1 |
|  | A mobile application | HP2 | 31 | 12 | 38.7 |
|  | Other: both appendix and hyperlinks; data export capacity; incorporate into clinical software; appendix with HP training options | HP2 | 31 | 4 | 16.1 |
|  | A Wiki system | HP2 | 31 | 1 | 3.2 |
| Presentation: consumer-friendly | The guideline should be written for consumers in lay language with a simple rationale for each recommendation | CS2 | 59 | 59 | 100 |
|  | I find diagrams such as flow-charts helpful to understand and learn new information | CS2 | 59 | 46 | 78.0 |
| Content: detail | Referral pathways with capacity for local adaptation would be clinically useful | HP2 | 31 | 29 | 93.5 |
|  | Information about how to get help for fatigue management in my local area would be useful | C2 | 59 | 54 | 91.5 |
|  | The guideline should contain links to additional detail about specific contributing factors such as medications, electrolyte levels, nutritional parameters and rate of physical activity change | HP2 | 32 | 28 | 90.3 |
|  | The guideline should contain links to additional detail about physical examinations and significance of findings | HP2 | 31 | 27 | 87.1 |
|  | An electronic checklist of contributing/risk factors for fatigue, with space to enter which assessments were performed, would be a useful clinical tool | HP2 | 32 | 21 | 65.6 |

**RESULTS: Theme 2 – Defined roles / knowledgeable health professionals**

| Sub-theme | Item or statement | Survey | N | Agree (n) | Agree (%) |  |
| --- | --- | --- | --- | --- | --- | --- |
| Health professional roles | I want to know which health professional is overseeing and monitoring my fatigue levels and supporting me | C2 | 59 | 58 | 98.3 | |
|  | A practice nurse (or other designated professional) could screen for tachycardia, shortness of breath and signs of nutritional deficiencies (oral) and anaemia (eyes) and refer to the appropriate professional for further assessment | HP2 | 31 | 30 | 96.8 | |
|  | Gait, posture, muscle wasting and range of motion would ideally be assessed by a relevant health professional; if appropriate | HP2 | 31 | 30 | 96.8 | |
|  | All HPs should be able to screen for fatigue | HP2 | 32 | 30 | 93.8 | |
|  | Determination of which HPs should take responsibility for assessments, interventions and follow up would improve consistency of practice | HP2 | 31 | 27 | 87.1 | |
| Health professional training | |  |  |  |  | |
| What is your preferred method/s of learning about assessment and management of CRF? | Interactive website | HP2 | 31 | 24 | 77.4 | |
|  | Workshop at conference |  | 31 | 17 | 54.8 | |
|  | Workshop / seminar at workplace |  | 31 | 16 | 51.6 | |
|  | Webinar, video conference |  | 31 | 15 | 48.4 | |
|  | Power point / static web page |  | 31 | 5 | 16.1 | |
|  | Local multidisciplinary community of practice |  | 31 | 1 | 3.2 | |
| What type of educational content do you prefer? | Both specific (interventions) and generic (guideline) | HP2 | 31 | 21 | 67.7 | |
|  | Specific on how to assess or deliver interventions |  | 31 | 9 | 29.0 | |
|  | Tailored / modules |  | 31 | 1 | 3.2 | |
|  | Generic about guideline use |  | 31 | 0 | 0 | |
| Consumer views | Make it real. I would like health professionals to know about how fatigue has affected real people like me | C2 | 59 | 57 | 96.6 | |
|  | On-line education about managing fatigue should be available to all health professionals involved in my cancer care | C2 | 59 | 55 | 93.2 | |

RESULTS: Theme 3 – Integrate with existing practices

| Sub-theme | Statement | Survey | N | Agree (n) | Agree (%) |
| --- | --- | --- | --- | --- | --- |
| Systems of care | Fatigue management should be a part of routine cancer services | C2 | 59 | 57 | 96.6 |
| Fatigue screening | Once clinicians identify moderate to severe fatigue they should seek advice and/or refer for comprehensive assessment | HP2 | 32 | 31 | 96.9 |
|  | Ask me about my fatigue level during routine appointments | C2 | 59 | 48 | 81.4 |
|  | Ask me about my fatigue level when asking about pain, nausea and other symptoms | C2 | 59 | 42 | 71.2 |
|  | Should fatigue screening be included in a multi-symptom checklist or a fatigue-specific tool?  Multi-symptom  Both  Fatigue-specific | HP2 | 32 | 17  12  3 | 53.1  37.5  9.4 |
| Assessment practices | Applying standardised diagnostic criteria for CRF is useful in the clinical setting to distinguish CRF from other types of fatigue | HP2 | 32 | 26 | 81.3 |
|  | A self-assessment for patient to identify issues would be time-efficient for clinicians | HP2 | 32 | 26 | 81.3 |
|  | A stratified approach to comprehensive assessment of CRF would be clinically feasible | HP2 | 32 | 22 | 68.8 |
|  | Would you use a guide to communicating with patients about fatigue e.g. ‘How to discuss fatigue’? | HP2 | 31 | 18 | 58.1 |
| Leadership support / endorsement | What are the five most important factors that would encourage you to adopt the CAPO CRF guideline?  #3 If service leaders, management or government endorsed the guideline  #5 If it were endorsed by a leading Australian cancer organisation | HP2  C2 | 35  32 | 22  18 | 62.9  56.3 |

RESULTS: Theme 4 – Consumer-focused care

| RESULTS: Theme 4 – Statement / Option | Survey | N | Agree (n) | Agree (%) |
| --- | --- | --- | --- | --- |
| Screening – Time point |  | | | |
| HP2: Which are essential time points for fatigue screening in Australia?  C2: From your perspective, when would be the best times to have fatigue screening? | | | | |
| At diagnosis  At diagnosis or start of treatment as baseline | HP2  C2 | 32  59 | 24  48 | 75.0  81.4 |
| At end of treatment  At end of a treatment course | HP2  C2 | 32  59 | 21  52 | 65.6  89.8 |
| At start of treatment cycle  During routine assessment before each new treatment | HP2  C2 | 32  59 | 20  52 | 62.5  88.1 |
| Change in condition  Changed phase of care  After hospitalisation or changed health status | HP2  HP2  C2 | 32  32  59 | 19  16  51 | 59.4  50.0  86.4 |
| 3 months post treatment | HP2  C2 | 32  59 | 18  50 | 56.3  84.7 |
| At each review  One month after treatment | HP2  C2 | 32  59 | 18  43 | 56.3  72.9 |
| 6 months post treatment | HP2  C2 | 32  59 | 17  43 | 53.1  72.9 |
| At annual check up | HP2  C2 | 32  59 | 12  48 | 37.7  81.4 |
| If previously screened positive  Patient / carer request | HP2  HP2 | 32  32 | 12  1 | 37.7  3.1 |
| Screening and assessment |  |  |  |  |
| It is essential to be made aware of the possibility of fatigue and how to measure it, when you are first diagnosed with cancer | C2 | 59 | 53 | 89.8 |
| It is important to me to have some say in when, where and how I am assessed if I have moderate to severe fatigue | C2 | 59 | 52 | 89.8 |
| I would like to be given the choice of doing a paper, electronic or verbal questionnaire to assess my fatigue | C2 | 59 | 50 | 88.8 |
| I would prefer a longer appointment for fatigue assessment compared to extra visits | C2 | 59 | 46 | 78.0 |
| What mode of questionnaire would you prefer to assess your fatigue?  No preference  Paper/pen  Electronic  Verbal / discussion  Combination of paper / verbal | C2 | 59 | 16  14  14  13  2 | 27.1  23.7  23.7  22.0  3.4 |
| Automatic bookings for non-essential appointments such as education or exercise might help me attend, especially if I am feeling tired | C2 | 59 | 32 | 54.2 |
| Education for consumers |  |  |  |  |
| Access to individual or group education about fatigue supported by written material is important to me | C2 | 59 | 53 | 89.8 |
| Information and education about fatigue should be offered at different levels of detail (e.g. basic, standard, detailed) | C2 | 59 | 53 | 89.8 |
| More detailed information about fatigue prevention can come once treatment has started. | C2 | 59 | 45 | 76.2 |
| What is your preferred format to receive educational material about fatigue?  Leaflet/printed material  Individual counselling  Group education sessions  Web Page  On-line interactive  DVD  Other | C1 | 32 | 19  18  10  9  3  1  2 | 59.4  56.3  31.3  28.1  9.4  3.1  6.2 |

RESULTS: SURVEY 1

What are the five most important factors that would encourage you to adopt the CAPO CRF guideline?

|  | Statement | Survey | N | Agree (n) | Agree (%) |
| --- | --- | --- | --- | --- | --- |
| HP#1 | If the procedures are easily put into practice | HP1 | 40 | 28 | 70.0 |
| HP#2 | Availability of staff educational materials or additional training in the guideline procedures | HP1 | 40 | 24 | 60.0 |
| HP#3 | If service leaders, management or government endorsed the guideline | HP1 | 40 | 22 | 55.0 |
| HP#4 | Availability of published evidence of the guideline's effectiveness | HP1 | 40 | 20 | 42.5 |
| HP#5 | If forms, resources and referral pathways were available electronically | HP1 | 40 | 17 | 42.5 |
| CS#1 | If my health professional promoted its use | C1 | 32 | 24 | 75.0 |
| CS#2 | If service leaders, management or government endorsed the guideline | C1 | 32 | 23 | 71.9 |
| CS#3 | Availability of health professionals with expertise to assess and treat CRF | C1 | 32 | 21 | 65.6 |
| CS#4 | If it were endorsed by a leading Australian cancer organisation | C1 | 32 | 18 | 56.2 |
| CS#5 | If fatigue is taken seriously by my health care providers | C1 | 32 | 18 | 56.2 |
